# A Case Report and Literature Review of Oligomeganephronia

**DOI:** 10.3389/fmed.2022.811992

**Published:** 2022-03-22

**Authors:** Xu-Hao Wang, Lei Pan, Shan He, De-Lei Kong, Wei Wang

**Affiliations:** Department of Respiratory and Critical Care Medicine, The First Hospital of China Medical University, Shenyang, China

**Keywords:** renal pathology, diagnosis, focal segmental glomerulosclerosis, renal insufficiency, oligomeganephronia

## Abstract

A 23-year-old woman was admitted to the hospital with proteinuria and mildly elevated creatinine, and a renal biopsy confirmed the diagnosis of oligomeganephronia (OMN). OMN is an extremely rare bilateral renal hypoplastic disease, and its diagnosis mainly relies on the pathological results obtained from renal biopsy. At present, there is no effective treatment for OMN. Here, we report a case with mild renal insufficiency and proteinuria as the main symptom and present a summary of the clinical characteristics of ON along with a review of the literature on OMN.

## Introduction

Oligomeganephronia (OMN) is a rare congenital bilateral renal hypoplasia disease, which was first reported by Royer et al. in 1962 (1) and is characterised histopathologically by a reduced number of nephrons and marked hypertrophy of the glomeruli. The onset of OMN is insidious and is often overlooked, as early clinical manifestations are non-specific and mainly present as proteinuria, increased nocturia, and polydipsia. All patients develop progressive renal failure early in the disease course, leading to end-stage renal disease. OMN is more frequently reported in children, with few cases reported in adults. To date, two forms of oligomeganephronia have been described in the literature (2): one of which is a solitary sporadic form characterised as follows: (1) congenital rather than genetic disease; (2) typically an isolated anomaly; and (3) reduced volume of both kidneys, hypertrophy, and reduction in the number of nephrons. The other form is associated with multiple congenital anomalies, and these patients are often born with multi-system dysplasia, such as renal dysfunction syndrome, branchiootorenal syndrome, and Wolf-Hirschhorn syndrome (3). It is often associated with genetic mutations, such as PAX2 mutations and chromosome 4 deletions. This form has been reported in twins and siblings (4). The diagnosis of OMN is based on pathological findings from renal biopsy. Clinically, there is still a lack of effective treatment for OMN, and comprehensive symptomatic treatment is currently the mainstay.

## Case Report

**Figure 1:**
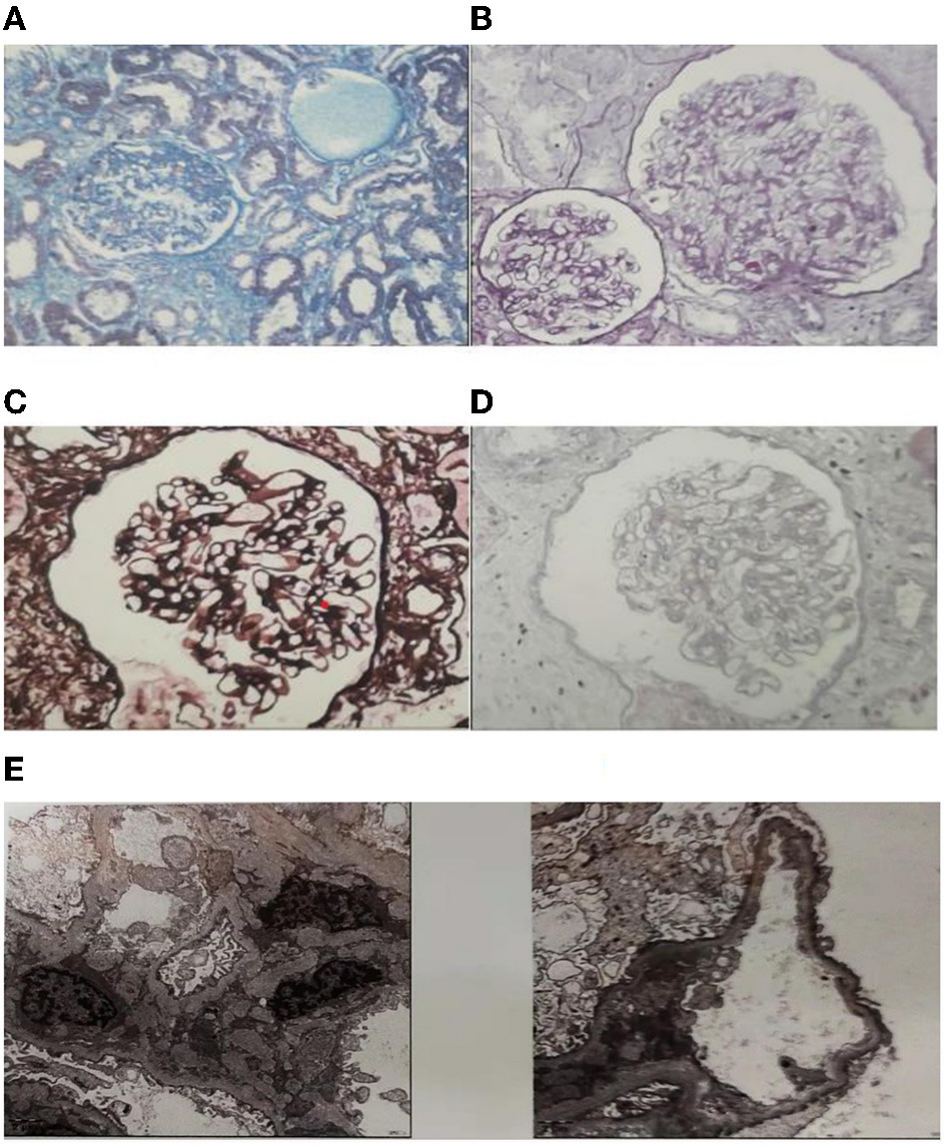
Histological features of the patient. **(A)** Segmental sclerosis with adhesions and podocyte hyperplasia, glomerular basement membrane flexure, wrinkling, cystic dilatation, Masson Stain. **(B)** Glomerular hypertrophy (~300 μm in diameter). The podocytes were swollen and had vacuolar degeneration, PAS Stain. **(C)** Mesangial cells and the mesangial matrix were mildly proliferated, mild focal fibrosis of renal interstitium, PASM Stain. **(D)** There was a granular, vacuolar formation of renal tubular epithelial cells and focal atrophy of the renal tubules with luminal narrowing. Protein casts were seen in the lumen, HE Stain. **(E)** Electron microscopy pathology: foot process effacement (podocyte fusion), and deposition of electron-dense material visible in the mesangial region.

A 23-year-old previously healthy female presented with renal insufficiency and proteinuria with normal growth and development and no family history of renal disease. Her physical examination findings were as follows: 170 cm in height, 50 kg in body weight, body temperature of 36.2°C, pulse rate of 96 beats/min, respiration rate of 20 breaths/min, and blood pressure of 127/85 mmHg. Her laboratory test results were summarised in Table 1. Urinalysis showed microhematuria and Minimalproteinuria. In the four items of urinary microprotein, the increase of urinary microalbumin and urinary transferrin was more significant. Biochemistry showed severe mild creatinine height (creatinine count: 144 μmol/L) and moderately decreased glomerular filtration rate (glomerular filtration rate count: 44.2 mL/min/1.73 m^2^). The current patient should be in stage 3 chronic kidney disease. Other Biochemistry indexs were slightly changed or normal.The ultrasound of both kidneys showed enhanced cortical echogenicity, good blood flow in both kidneys, a left kidney cyst, and left and right kidney sizes of 9.6 × 3.7 × 3.5 cm and 9.4 × 3.5 × 3.6 cm, respectively. The electrocardiogram, cardiac Doppler ultrasound, and lung computed tomography did not reveal any significant abnormalities.Renal biopsy revealed (Figure 1): a significantly reduced number of glomeruli as compared to normal, glomerular hypertrophy (~300 μm in diameter), segmental sclerosis with adhesions and podocyte proliferation, proliferation of mesangial cells and mesangial matrix, and deposition of electron-dense material (C3 and IgM) visible in the mesangial region. These were consistent with the known pathological manifestations of OMN.

**Table 1:** Laboratory data of the patient.

**Urinalysis**		**Biochemistry**	
Occult blood	1+	Blood creatinine	144 μmol/L (41–73 μmol/L)
Protein	3+	Serum cystatin C assay	1.61 mg/L (0.53–0.95 mg/L)
Urine protein assay	28.6 mg/dL (1.00–14.00 mg/dL)	Glomerular filtration rate	44.2 mL/min/1.73 m^2^
Proteinuria in 24-h urine	0.629 g/24 h (0.028–0.141 g/24 h)	Serum uric acid	385 μmol/L (155–357 μmol/L)
Urinary β2 microglobulin	1.17 mg/L (0.00–0.23 mg/L)	Glycoantigen assay (CA199)	31.1 U/mL (0.00–27.00 U/ml)
Urinary α1 microglobulin	14.8 mg/L (0.00–12.00 mg/L)	25 hydroxyl Vitamin D3	8.37 ng/mL (11.10–42.90 ng/mL)
Urinary microalbumin	850 mg/L (0.00–30.00 mg/L)	Triglycerides	1.79 mmol/L (0.00–1.70 mmol/L)
Urinary transferrin	23.6 mg/L (0.00–2.41 mg/L)	Serum thyroid stimulating hormone	5.0 mlU/L (0.35–4.94 mlU/L)
Urinary IgG	35 mg/L (0.00–9.60 mg/L)		
Urinary free light chain KAPPA	105 mg/L (0.00–25.80 mg/L)		
Urinary free light chain LAMBDA	28.1 mg/ L (0.00–11.3 mg/L)		

*Routine blood test findings, blood ions, liver function were normal, four infectious diseases (hepatitis B antigens, hepatitis C antibodies, acquired immunodeficiency syndrome antibodies, and syphilis antibodies), The urine paraprotein assay, serum protein electrophoresis, immunofixation electrophoresis, immunoglobulin complete set, complements (C3 and C4), IgG4 subtypes, T-cell subsets, rheumatic antibody series, anti-neutrophil cytoplasmic antibodies, anti-glomerular basement membrane antibodies, and anti-phospholipase A2 receptor antibodies were normal*.

Based on the patient's clinical presentation, examination data, and biopsy results, the patient was ultimately diagnosed with oligomeganephronia (OMN). The patient was advised to take losartan 50 mg orally once daily.

## Literature Review

We used PubMed® and the China National Knowledge Infrastructure to retrieve reports on OMN from 1970 to 2020, and a total of 39 complete cases were included. The 39 cases were summarised and reviewed below, and all cases were shown in the Appendix in Supplementary Material.

OMN can be broadly classified into two categories. One is a solitary sporadic form, in which the onset is often during adolescence. There were 30 patients in this category, with the age of onset ranging from 2 to 45 years and a mean age of (23.5 ± 9.5) years. There were 23 male and 7 female patients with OMN, with a male-to-female ratio between 3:1. We found that only a few patients were low-birth-weight infants or patients with previous chondrosarcoma, unexplained fever, and/or growth retardation; the rest of the patients were previously healthy. Most patients with the solitary sporadic form of OMN have an insidious onset, with a duration of disease ranging between 1 month and 15 years.The course of disease is more than 1 year in 50% of patients. Clinical manifestations often include proteinuria, elevated creatinine, dysuria, increased nocturia, and hypertension. In terms of extrarenal damage, four patients were found to have hypertension, and one patient had a large optic disc in both eyes. In terms of laboratory findings, 25 cases (83.3%) had elevated creatinine, and proteinuria was present in almost all the patients. As far as ultrasound findings were concerned, bilateral renal atrophy was present in 25 (83.3%) patients, and unilateral renal atrophy was present in 1 patient. In terms of pathological findings, the solitary sporadic form of OMN often manifests as sparse glomeruli with an increased glomerular diameter and with a mean glomerular diameter of 224.32 ± 67.62 μm. There were 9 patients with concomitant focal segmental glomerular sclerosis, 17 patients with IgM deposition, 1 patient with IgG deposition, and 16 patients with concomitant podocyte fusion. Mutation of the PAX2 gene was found in only one patient.

In terms of treatment, 12 out of 30 patients were reported and followed up, 10 of whom used pharmacological treatment with the following regimens: angiotensin-converting-enzyme inhibitors (ACEIs) combined with angiotensin II receptor blockers (ARBs), calcium channel blockers (CCBs), α receptor blocker, β receptor blocker, and aspirin; ACEI combined with ARB and CCB; sole treatment with ARB; sole treatment with ACEI; ACEI combined with statins; hormone therapy combined with ACEI; sodium bicarbonate combined with aluminium hydroxide gel and antibiotics; sole treatment with Chinese medicine; and haemodialysis combined with kidney transplantation, respectively. These patients were followed up for 2 months to 5 years. The creatinine increased three-fold within 5 years in the patient receiving Chinese medicine; two patients were solely treated with ARB-type drugs; creatinine did not change significantly in one patient after 1.5 years of follow-up, and creatinine was increased in one patient after 2 years of follow-up, considering that the latter may have been associated with a high 24-h urine protein result. Meanwhile, there were two patients with elevated 24-h urine protein >1 g. One of these patients with concomitant focal segmental glomerular sclerosis was treated with ACEI in combination with statins and had a 1.27-fold increase in creatinine at the 5-year follow-up, and the other patient was treated with hormones in combination with ACEI drugs and had no significant change in creatinine at the 1.5-year follow-up. The patient who was solely treated with Chinese medicine had a 3.37-fold increase in creatinine after 5 years. Renal transplant patients remained in good health within 2 years (Table 2).

**Table 2:** Summary of the solitary sporadic form of OMN.

**Variable**	**Category**	**Case, *n* (%)**
Sex	Male Female	23 (76%) 7 (23%)
Mean age, years (range)		23.5 ± 9.5
Body mass index (kg/m2)	<18.5 >18.5 unknown	10 (33%) 8 12
low birth weight (yes or no)	Yes No Unknown	3 (10%) 7 (23%) 20 (66.7%)
Past history	Past history health recurrent fever Chondroma developmental retardation	27 (90%) 1 1 1
Family history	Father, ESRD;sister, MPGN brother, ureter-too-short;great-uncle, three kidney Yes (Concrete is unknown) No	1 1 2 26 (85%)
blood pressure (mmHg)	Hypertension Normal	4 (15%) 26 (85%)
Initial symptoms	Hypertension Proteinuria Right flank pain Dysuria Gastrointestinal discomfort Fever polyuria Elevated sera Creatinine Edoema of face and limbs	1 20 (66.7%) 1 1 1 2 12 (40%) 3 1
Extrarenal damage	Large optic disc, high frequency hearing loss No	1 29
24-h urine protein (g/day)	<1 1–2 >2	16 (53.3%) 7 (23.3%) 5 (16.7%)
serum creatinine	elevated creatinine Normal	25 (83%) 5 (16.7%)
Kidney ultrasound	Bilateral renal atrophy Right renal atrophy Normal Unknown	25 (83%) 1 3 1
Pathological	Glomerular diameter (μm) Focal segmental glomerulosclerosis IgM deposition IgG deposition podocyte fusion	(224.32 ± 67.62) μm 9 (30%) 19 (63.3%) 1 19 (63.3%)
Gene mutation	PAX2 Gene mutation	1
course of diease		1 months−15 years
Medication	ACEI, ARB, CCB, αβblocker, aspirin ARB ACEI, Atorvastatin sodium bicarbonate, aluminium hydroxide gel, antibiotic Hemodialysis, renal transplant ACEI traditional chinese medicine	1 2 2 1 1 2 1
Outcome	Creatinine is higher than before	3
	No change in creatinine	7

The other form is associated with multiple congenital anomalies, with a total of nine cases in this summary presenting with symptoms at or shortly after birth. Patients with the second category were often associated with poor developmental growth and physical deformities, and nearly 55% of the patients had low birth weight. The clinical manifestations of OMN associated with congenital anomalies commonly include oliguria and proteinuria, and it is most often accompanied by extrarenal damage, such as facial deformities, limb deformities, respiratory failure, and cardiac failure. Elevated creatinine was reported in only three patients, and all the patients had small kidneys. Patients with congenital anomaly-related OMN are often associated with genetic mutations. Five patients had Wolf-Hirschhorn syndrome (partial deletion of chromosome 4), and two patients were diagnosed with limbic renal syndrome. Moreover, this group of patients have a low survival rate and rapid disease progression. In neonatal-onset patients, the maximum follow-up survival time was 27 months. In two adolescent-onset patients, a two-fold increase in creatinine was reported 3–4 years after treatment with ACEI/ARB (Table 3).

**Table 3:** Summary of OMN associated with congenital anomalies.

**Variable**	**Category**	**Case**
Sex	Male	9,
	Female	0
Mean age, years (range)		0
Body mass index (kg/m^2^)	<18.5	3
	>18.5	1
	Unknown	5
low birth weight (yes or no)	Yes	5
	No	4
Past history	Intrauterine retardation, mild mental retardation, mild language retardation	1
	Intrauterine growth delay, type III osteogenesis imperfectan	1
Family history	brother;multiple malformations, kidneys atrophy	1
	father and brother;Chromosomal abnormalities, 46, XY, t (4:6) (p12, p23)	1
	Male twins died in utero	1
	No	6
blood pressure (mmHg)	Normal	3
	Unknown	6
Initial symptoms	Proteinuria	1
	Dysuria	1
	Polyuria	4
	Seizures, hypotonia, gastroesophageal reflux, with specific anatomical features	1
	Limbs deformity	1
	Lethargy, severe vision loss, limb loss	
Extrarenal damage	Facial deformity	1
	Limbs deformity	1
	Multiple malformations	4
	Heart failure, respiratory failure	2
	Severe vision loss, limb loss	1
	Severe vision loss, limb loss	1
24-h urine protein (g/day)	0.45–0.56	1
	Unknown	8
Serum creatinine	Elevated creatinine	5
	Normal	1
	Unknown	3
Kidney ultrasound	Bilateral renal atrophy	6
Pathological	Glomerular diameter (μm)	87.5–350 μm
	Focal segmental glomerulosclerosis	2
	IgM deposition	1
	Podocyte disappeared	1
Gene mutation	Acrorenal syndrome	2
	Wolf-hirschhorn syndrome, partial deletion of chromosome 4	5
	Unknown	2
course of diease		1 days−6 years
Medication	ACEI	1
	ARB	1
	Correct acidosis, vitamin D	1
Outcome	Creatinine is higher than before	2
	Died	4
	Unknown	3

## Discussion

We conducted the first systematic review of previous OMN cases and compared OMN patients into two groups, the solitary sporadic form of OMN and OMN associated with congenital anomalies. However, because this disease is relatively rare and related reports span a long period of time, the relevant information is not perfect. OMN is characterised by a decrease in the number of nephrons and a marked hypertrophy of the glomeruli. A maximum reduction of 80% in the number of nephrons and hypertrophy of the remaining nephrons with glomerular volume reaching 12–15 times the normal volume and tubular volume reaching 17 times the normal volume has been reported (5, 6). Globle studies have shown a male predisposition to the disease with a male-to-female ratio of 3:1 (4), while a male-to-female ratio between 4:1 and 5:1 was observed in our all analysis. But Th count is similar to the result for the solitary sporadic form of OMN. Its aetiology is unclear, and studies have suggested that it may be associated with intrauterine growth retardation, low birth weight, prematurity, and poor intrauterine developmental growth related to some genetic mutations, such as PAX2 mutation, EAY1 mutation, RET1 mutation, and chromosome 4 deletion syndrome (7–11). It is well known that the full number of nephrons is established up to the period of the first 32 or even 36 weeks of gestation, without the creation of new nephrons after that period. However the information about prenatal anamnesis regarding possible toxic exposure or other insults during the pregnancy was often ignored by doctors or patients. In our review summary, there were seven cases of low-birth-weight infants, two cases of intrauterine growth retardation, one case of PAX2 mutation, five cases of Wolf-Hirschhorn syndrome (partial deletion of chromosome 4), and two patients diagnosed with limbic renal syndrome. Salomon et al. analysed three patients with detectable PAX2 mutation (12); these patients also had ocular abnormalities. However, none of the patients with PAX2 mutation in this review reported concomitant extrarenal damage.

In 1997, Broyer et al. established strict diagnostic criteria for OMN (6): (1) the sum of the length of both kidneys is <80% of the length of one kidney in healthy children of the same age; (2) the glomerular filtration rate is reduced to 30% of the normal rate; and (3) there is no urinary tract malformation or evidence of significant vesicoureteral reflux. However, many subsequent reports have not used these criteria. A renal biopsy is required for the diagnosis of this disease. Glomerular hypertrophy must be determined by measuring the diameter of the glomeruli, and glomerular scarcity is defined as <10 glomeruli in the cortical region following standard histological sampling. Chen Huiping et al. summarised the main histologic manifestations (13), including: (1) a low number of glomeruli (when avoiding the simple medulla and medullary line) and <10 glomeruli in the cortical tissue, mostly between two to six; (2) an increase in glomerular volume, the diameter of which is twice or more than twice that of normal human glomeruli (some studies have reported that the diameter can be more than three times that of the normal human glomeruli); (3) adjacent glomeruli with hypertrophy of the proximal tubule; and (4) thickening of the glomerular Bowman's capsule wall.

Most patients develop clinical manifestations, such as anorexia, vomiting and dehydration, polyuria, and polydipsia in the first 2 years of life. However, these clinical manifestations are often seen in patients with congenital anomalies related to OMN, and to date, only one patient with a solitary sporadic form of OMN presented with recurrent fever in infancy. As the sparse glomeruli become chronically overloaded, the glomerular volume increases, leading to glomerular basement membrane damage, which in turn results in hypertension, haematuria, proteinuria, and decreased renal function. Urinary protein levels also gradually increase with progressive renal failure. Approximately 62.5% of patients develop proteinuria with concomitant impaired renal tubular function before the age of 20 years (3); 11 patients (28%) in the existing literature had proteinuria as the initial symptom. Almost all patients had proteinuria, and only four (10%) patients had hypertension. The disease was previously reported to be frequently combined with focal segmental glomerular sclerosis (FSGS), and research by McGraw et al. (14) showed that OMN patients had significant proteinuria and FSGS and were associated with a rapid decline in renal function. FSGS was reported in 11 patients in this analysis, and no patients with significantly elevated creatinine were seen in the follow-up group. Both kidneys were of a reduced size on ultrasound, with some patients having normal kidney sizes, and 34 patients (~94%) presenting with reduced kidney sizes.

In the solitary form of OMN, nearly 40% (12/30) of patients have developmental problems, including low BMI, low birth weight, and developmental delay. It is possible that even these patients may have other developmental malformations of organs or limbs, which may not be recognised because they do not have typical characteristics or have less impact on quality of life. Therefore, compared with patients with OMN associated with congenital anomalies, the early clinical manifestations of the solitary form of OMN are less and not easily detected, and the disease progresses more slowly.

At present, there is no effective treatment for OMN, and the main clinical focus is on comprehensive symptomatic treatment. The usage of anti-hypertensive treatment with ACEI/ARB may help to reduce glomerular hyperfiltration, and, hence, delay OMN progression. However, for patients with high urinary protein and focal segmental glomerular sclerosis, the effect of treatment with ACEI/ARB drugs alone is limited, and combinational therapy with hormone or statins is recommended. However, it will eventually progress to end-stage renal disease, and long-term replacement therapy or early renal transplantation may be a better treatment modality.

## Conclusion

In summary, OMN is a congenitally dysplastic disease of both kidneys, and the diagnosis of which relies on pathological biopsy. Congenital anomaly-associated OMN often develops shortly after birth, with extrarenal damage, such as physical deformities, and is often associated with genetic mutations, poor drug treatment efficacy, and low survival rates. The clinical manifestations of the solitary sporadic form of OMN are non-specific and often overlooked. Some patients have hypertension and ocular changes, and the use of ACEI/ARB treatment may delay the progression of OMN, though it induces elevated urinary protein. Combinational therapy with drugs, such as hormones or statins, may be effective. Early renal transplantation may be a better treatment modality.

## Author Contributions

X-HW and LP contributed to conception and design of the study. SH organized the database. X-HW and SH performed the statistical analysis. X-HW wrote the first draft of the manuscript. LP, SH, D-LK, and WW wrote sections of the manuscript. All authors contributed to manuscript revision, read, and approved the submitted version.

## Conflict of Interest

The authors declare that the research was conducted in the absence of any commercial or financial relationships that could be construed as a potential conflict of interest.

## Publisher's Note

All claims expressed in this article are solely those of the authors and do not necessarily represent those of their affiliated organizations, or those of the publisher, the editors and the reviewers. Any product that may be evaluated in this article, or claim that may be made by its manufacturer, is not guaranteed or endorsed by the publisher.
