# Supplementary material for: A Case Report and Literature Review of Oligomeganephronia
Source: Front Med (Lausanne). 2022 Mar 22;9:811992. doi: 10.3389/fmed.2022.811992 (PMC8980273; doi:10.3389/fmed.2022.811992)
Supplement: Supplementary file 1 [file Data_Sheet_1.docx]

Appendix

Table1 Summary of the solitary spotadic form of OMN

| Num-ber | Author | Year | Age（years） | Sex | low birth weight | Past history | Family history | BMI (kg/m2) | blood pressure (mmHg) | Initial symptoms | Extrarenal damage | course of diease(years) | 24-h urine protein (g/day) | serum creatinine（µmol/L） | Kidney ultrasound | Pathological | Glomerular diameter(μm) | Focal segmental glomerulosclerosis | Fluorescent staining | Electron microscopic examination | Gene mutation | Medication | Follow-up time | Follow-up creatinine (µmol/L) |
| --- | --- | --- | --- | --- | --- | --- | --- | --- | --- | --- | --- | --- | --- | --- | --- | --- | --- | --- | --- | --- | --- | --- | --- | --- |
| 1 | Kawanishi, K, et al | 2011 | 36 | male | normal | Past history health | no | 20.9 | 166/113 | hypertension during physical examination | NO | unknown | 0.18 | 234.26 | Bilateral renal atrophy |  | 200 | no | IgM deposition | podocyte fusion | unknown | ACEI、ARB、CCB、αβblocker, aspirin | 3.5 years | 221-265.2 |
| 2 | Kawanishi, K, et al | 2011 | 19 | female | low birth weight | Past history health | no | 21.8 | normal | proteinuria | NO | unknown | 0.53 | 100.77 | Bilateral renal atrophy |  | 310 | yes | IgM deposition | podocyte fusion | unknown | ACEI、ARB、CCB | 3 years | 97.24-114.92 |
| 3 | Kawanishi, K, et al | 2011 | 21 | male | normal | Past history health | Father,ESRD;sister,MPGN | 19.1 | normal | proteinuria | NO | unknown | 0.65 | 106.08 | normal |  | 270 | no | IgM deposition | normal | unknown | ARB | 1.5 years | 97.24-106.08 |
| 4 | Bito, L,et al. | 2020 | 23 | female | unknown | Past history health | no | unknown | normal | proteinuria | NO | 7 | 2.5 | 145 | normal |  | 268 | yes | IgM deposition | podocyte fusion | PAX2 Gene mutation | ACEI、Atorvastatin | 5 years | 185 |
| 5 | Fuke, Y., et al | 2012 | 23 | male | normal | Past history health | no | 24.95 | 160/94 | proteinuria | NO | 7 | 3.86 | 138.7 | Bilateral renal atrophy |  | 325 | yes | unknown | podocyte fusion | unknown | ARB | 2 years | 150-203 |
| 6 | Alves, R.J, et a | 2012 | 33 | male | unknown | Past history health | no | unknown | normal | right flank pain | NO | unknown | 2.9 | 172.38 | Bilateral renal atrophy |  | unknown | no | IgM deposition | unknown | unknown | no | no | no |
| 7 | Abdelraheem, M,et al | 2004 | 14 | male | unknown | Past history health | no | Height in the 93rd percentile,weight in the 50th percentile | normal | dysuria | NO | unknown | unknown | unknown | Bilateral renal atrophy |  | unknown | no | unknown | unknown | unknown | no | no | no |
| 8 | Adelman, R.D. and S. Shapiro | 1977 | 2 | female | normal | recurrent fever | no | 15.18 | normal | unknown | NO | unknown | 0.204 | 282.88～327.08 | Bilateral renal atrophy |  | unknown | no | unkonwn | unknown | unknown | sodium bicarbonate、aluminium hydroxide gel、antibiotic | 2 months | 150.28 |
| 9 | Carter, J.E. and D.S. | 1970 | 16 | male | unknown | Chondroma | no | 16.7 | normal | gastrointestinal discomfort,fever,proteinuria, | NO | 15 | 1.046 | 123.76 | unknown |  | 250-300 | no | IgG deposition | no | unknown | no | no | no |
| 10 | Morita, T., et al | 1973 | 12 | male | low birth weight | developmental retardation | no | 15.02 | normal | proteinuria | NO | 9 | 0.471 | 132.6 | Bilateral renal atrophy |  | 260 | unknown | unknown | unknown | unknown | no | no | no |
| 11 | Janin-Mercier, A., et al | 1985 | 11 | male | unknown | Past history health | no | 19.44 | normal | polyuria,proteinuria, | NO | unknown | 0.98 | 220 | Bilateral renal atrophy |  | 300 | yes | IgM deposition | no | unknown | Hemodialysis、renal transplant | 2年，身体良好 | no |
| 12 | Lam, M., et al | 1982 | 11 | male | normal | Past history health | brother,ureter too short;great-uncle,three kidney | 14.49 | normal | proteinuria | NO | 3 | microalbuminuria | 114.92 | Right renal atrophy |  | 225 | no | IgM deposition | no | unknown | no | no | no |
| 13 | Yang, X.D., et al | 2014 | 26 | male | normal | Past history health | no | unknown | normal | proteinuria | NO | 2 | unknown | 160 | Bilateral renal atrophy |  | 305 | no | unknown | unknown | unknown | no | no | no |
| 14 | Huiping Chen, et al | 2007 | 22 | male | unknown | Past history health | no | 16.8 | normal | elevated sera creatinine | NO | unknown | 1.19 | 129.1 | Bilateral renal atrophy |  | 293.1 | no | IgM deposition | podocyte fusion | unknown | no | no | no |
| 15 | Zheng Yandan et al. | 2019 | 28 | female | normal | Past history health | no | unknown | normal | elevated sera creatinine | NO | unknown | 0.16 | 139 | Bilateral renal atrophy |  | 227 | no | IgM deposition | no | unknown | no | no | no |
| 16 | Zhang Wenjing et al. | 2020 | 18 | male | normal | Past history health | no | 16.18 | normal | proteinuria、elevated sera creatinine | NO | unknown | 0.48 | 162 | normal |  | 292.3 | no | IgM deposition | no | unknown | no | no | no |
| 17 | Lin Lirong et al | 2012 | 45 | male | unknown | Past history health | no | 16.73 | normal | edema of face and limbs | NO | unknown | 0.14 | 107.8 | Bilateral renal atrophy |  | 276.4 | no | IgM deposition | podocyte fusion | unknown | no | no | no |
| 18 | Zhang Liang et al | 2014 | 10 | male | normal | Past history health | yes | 19.38 | normal | 3 polyuria，2 proteinuria | NO | 1 | 1.44 | 96 | Bilateral renal atrophy |  | 230.08 | no | 1 case is IgM deposition | podocyte fusion | unknown | hormone、ACEI | 1.5 years | 103 |
| 19 | Zhang Liang et al | 2014 | 8 | male | normal | Past history health | no | 14.109 | normal |  | NO | 2 | 0.54 | 124 | Bilateral renal atrophy |  |  | no |  | podocyte fusion | unknown | ACEI | 2.5 years | 130 |
| 20 | Zhang Liang et al | 2014 | 4 | female | normal | Past history health | no | 22.22 | normal |  | NO | 2 | 0.67 | 78 | Bilateral renal atrophy |  |  | no |  | podocyte fusion | unknown | hormone、ACEI | 2 years | 72 |
| 21 | Zhang Liang et al | 2014 | 8 | male | normal | Past history health | yes | 15.82 | normal |  | NO | 1 | 0.17 | 86 | Bilateral renal atrophy |  |  | no |  | podocyte fusion | unknown | ACEI | 1 years | 91 |
| 22 | Zhang Liang et al | 2014 | 3 | female | normal | Past history health | no | 13.88 | normal |  | NO | 1 | 0.42 | 258 | Bilateral renal atrophy |  |  | no |  | podocyte fusion | unknown | traditional chinese medicine | 5 years | 870 |
| 23 | Wang Shaofan et al | 2009 | 9 | male | normal | Past history health | no | unknown | normal | polyuria，respiratory infection，fever,proteinuria | NO | 3 | 0.79 | 92.82 | Bilateral renal atrophy |  | 227 | yes | IgM deposition | podocyte fusion | unknown | unknown | no | no |
| 24 | Wang Shaofan et al | 2009 | 22 | male | normal | Past history health | no |  | hypertension，unknown value |  | NO | 1月 | 2.19 | 129.06 | Bilateral renal atrophy |  | 224.58 | no | no | podocyte fusion | unknown | unknown | no | no |
| 25 | Wang Shaofan et al | 2009 | 5 | male | normal | Past history health | no |  | hypertension，unknown value |  | large optic disc,high frequency hearing loss | 5 | 2.64 | 96.36 | Bilateral renal atrophy |  | 218.09 | yes | IgM deposition | podocyte fusion | unknown | unknown | no | no |
| 26 | Wang Shaofan et al | 2009 | 21 | male | normal | Past history health | no |  | normal |  | NO | 8 | 1.25 | 197.13 | Bilateral renal atrophy |  | 243.54 | yes | IgM deposition | podocyte fusion | unknown | unknown | no | no |
| 27 | Wang Shaofan et al | 2009 | 27 | female | normal | Past history health | no |  | normal |  | NO | 3 months | 1.13 | 139.67 | Bilateral renal atrophy |  | 228.5 | yes | IgM deposition | podocyte fusion | unknown | unknown | no | no |
| 28 | Wang Shaofan et al | 2009 | 11 | male | normal | Past history health | no |  | normal |  | NO | 6 | 1.15 | 95.47 | Bilateral renal atrophy |  | 220.55 | no | IgM deposition | podocyte fusion | unknown | unknown | no | no |
| 29 | Wang Shaofan et al | 2009 | 10 | male | low birth weight | Past history health | no |  | normal |  | NO | 1.5 | 0.89 | 81.33 | Bilateral renal atrophy |  | 204.37 | yes | IgM deposition | podocyte fusion | unknown | unknown | no | no |
| 30 | Wang Shaofan et al | 2009 | 15 | male | normal | Past history health | no |  | normal |  | NO | 5 months | 1.5 | 117.57 | Bilateral renal atrophy |  | 220.23 | no | no | podocyte fusion | unknown | unknown | no | no |

**Table 2** Summary of OMN associated with congenital anomalies

| Num-ber | Author | Year | Age（years） | Sex | low birth weight | Past history | Family history | BMI (kg/m2) | blood pressure (mmHg) | Initial symptoms | Extrarenal damage | course of diease(years) | 24-h urine protein (g/day) | serum creatinine（µmol/L） | Kidney ultrasound | Pathological | Glomerular diameter(μm) | Focal segmental glomerulosclerosis | Fluorescent staining | Electron microscopic examination | Gene mutation | Medication | Follow-up time | Follow-up creatinine (µmol/L) |
| --- | --- | --- | --- | --- | --- | --- | --- | --- | --- | --- | --- | --- | --- | --- | --- | --- | --- | --- | --- | --- | --- | --- | --- | --- |
| 1 | Gatto, A., et al | 2018 | 9 years | Male | normal | Intrauterine retardation, mild mental retardation, mild language retardation | NO | 17.5 | 130/80 | seizures, hypotonia, gastroesophageal reflux, with specific anatomical features | Facial deformity | 6 years | unknown | 92.82 | Bilateral renal atrophy |  | unknown | yes | IgM deposition | unknown | Wolf-hirschhorn syndrome, partial deletion of chromosome 4 | ACEI | 4 years | 176.8 |
| 2 | Konomoto, T., et al | 2017 | 16 years | Male | normal | Intrauterine growth delay, type III osteogenesis imperfecta | NO | 19.44 | unknown | proteinuria | NO | 5 years | unknown | 53.92 | Bilateral renal atrophy |  | 250-350μm | unknown | unknown | Podocyte disappeared | unknown | ARB | 3 yesrs | 112.26 |
| 3 | Miltenyi, M., et al | 1984 | 11days | Male | normal | NO | NO | 11 | 60/50 | Limbs deformities | Limbs deformities | 1days | 0.45-0.56 | 255 | Bilateral renal atrophy |  | unknown | unknown | unknown | unknown | Acrorenal syndrome | Correct acidosis, vitamin D | died ;27 months later | 435 |
| 4 | Al, S.A., et al | 1996 | 5years | Male | normal | NO | NO | Height and weight less than 3 percent | normal | Lethargy, severe vision loss, limb loss | severe vision loss, limb loss | unknown | unknown | 560 | Bilateral renal atrophy |  | 240 | yes | unknown | unknown | Acrorenal syndrome | unknown | unknown | unknown |
| 5 | Kusuyama, Y., et al | 1985 | newborn | Male | low birth weight | NO | NO | unknown | unknown | polyuria | Multiple malformations, heart failure, respiratory failure | 1days | unknown | unknown | Bilateral renal atrophy |  | 116.2±17.6 | unknown | unknown | unknown | Wolf-hirschhorn syndrome, partial deletion of chromosome 4 | unknown | died ;1 week later | unknown |
| 6 | Kusuyama, Y., et al | 1985 | newborn | Male | low birth weight | NO | brother;multiple malformations、kidneys atrophy | unknown | unknown | polyuria | Multiple malformations, heart failure, respiratory failure | 1days | unknown | unknown | Bilateral renal atrophy |  | 87.5±12.8 | unknown | unknown | unknown | Wolf-hirschhorn syndrome, partial deletion of chromosome 4 | unknown | died ;1 week later | unknown |
| 7 | Park, S.H. and J.G. Chi | 1993 | 4 months | Male | low birth weight | NO | unknown | unknown | unknown | polyuria | Multiple malformations | unknown | unknown | 97.24 | Bilateral renal atrophy |  | 141 | unknown | unknown | unknown | Wolf-hirschhorn syndrome, partial deletion of chromosome 4 | unknown | unknown | unknown |
| 8 | Park, S.H. and J.G. Chi | 1993 | newborn | Male | low birth weight | NO | Chromosomal abnormalities in father and brother.46,XY, t(4:6) (p12,p23) | unknown | unknown | dyspnea | Multiple malformations | 1days | unknown | unknown | Bilateral renal atrophy |  | 120.3 | unknown | unknown | unknown | Wolf-hirschhorn syndrome, partial deletion of chromosome 4 | unknown | unknown | unknown |
| 9 | Moerman, P., et al | 1984 | newborn | Male | low birth weight | NO | Male twins died in utero | unknown | unknown | polyuria | Bone rickets, fractures | 1days | unknown | 433.16 | Bilateral renal atrophy |  | unknown | unknown | unknown | unknown | unknown | unknown | died ;15 months later | 1043.12 |

REFERENCES:

1. Kawanishi K, Takei T, Kojima C, Moriyama T, Sugiura H, Itabashi M, Tsukada M, Uchida K, Honda K, Nitta K. Three cases of late-onset oligomeganephronia. NDT Plus. 2011 Feb;4(1):14-6. doi: 10.1093/ndtplus/sfq175.
2. Bitó L, Kalmár T, Maróti Z, Turkevi-Nagy S, Bereczki C, Iványi B. PAX2 Mutation-Related Oligomeganephronia in a Young Adult Patient. Case Rep Nephrol Dial. 2020 Nov 30;10(3):163-173. doi: 10.1159/000510841.
3. Fuke Y, Hemmi S, Kajiwara M, Yabuki M, Fujita T, Soma M. Oligomeganephronia in an adult without end stage renal failure. Clin Exp Nephrol. 2012 Apr;16(2):325-8. doi: 10.1007/s10157-011-0560-8.
4. Alves RJ, Oppermann K, Schein LE, Pêgas KL. A case of late-onset oligomeganephronia. J Bras Nefrol. 2012 Oct-Dec;34(4):392-4. doi: 10.5935/0101-2800.
5. Abdelraheem M, Watson AR, McCulloch TA. Oligomeganephronia: an unexpected cause of chronic renal failure. Saudi J Kidney Dis Transpl. 2004 Jan-Mar;15(1):53-6.
6. Adelman RD, Shapiro S. Bilateral renal hypoplasia with oligomeganephronia. Urology. 1977 May;9(5):571-5. doi: 10.1016/0090-4295(77)90259-x.
7. Carter JE, Lirenman DS. Bilateral renal hypoplasia with oligomeganephronia. Oligomeganephronic renal hypoplasia. Am J Dis Child. 1970 Dec;120(6):537-42. doi: 10.1001/archpedi.
8. Morita T, Wenzl J, McCoy J, Porch J, Kimmelstiel P. Bilateral renal hypoplasia with oligomeganephronia: quantitative and electron microsopic study. Am J Clin Pathol. 1973 Jan;59(1):104-12. doi: 10.1093/ajcp/59.1.104.
9. Janin-Mercier A, Palcoux JB, Gubler MC, de Latour M, Dalens H, Fonck Y. Oligomeganephronic renal hypoplasia with tapetoretinal degeneration. Report of one case with ultrastructural study of the renal biopsy. Virchows Arch A Pathol Anat Histopathol. 1985;407(4):477-83. doi: 10.1007/BF00709994.
10. Lam M, Halverstadt D, Altshuler G, Wenzl JE. Congenital oligomeganephronia in a solitary kidney: report of a case. Am J Kidney Dis. 1982 Mar;1(5):300-1. doi: 10.1016/s0272-6386(82)80029-2.
11. Yang XD, Shi W, Li D, Peng T. Oligomeganephronia: case report and literature review. Srp Arh Celok Lek. 2014 Nov-Dec;142(11-12):732-5. doi: 10.2298/sarh1412732y.
12. Huiping Chen, Shijun Li, Zhihong Liu, Oligomeganephronia [J]. Journal of Nephrology and Dialysis kidney Transplantation, 2007(02): 192-195.
13. Yandan Zheng, Yayao Li, Xiaohan Lu, Ping Gao. A case study of glomerular giant and rare disease [J]. J clin nephrology,2019,19(03):223-224.
14. Wenjing Zhang, Chao Zhang, Ping Lan, Jiping Sun. Glomerular hyperplasia: a case report [J]. Ann hui med,2020,41(02):232-233.
15. Lirong Lin, Jianguo Zhang, Jie Yang, et al. Glomerular hyperplasia: a case report [J]. Journal of the Third Military Medical University, 2012,34(01):80+89. doi:10.16016/j.1000-5404.2012.01.016.
16. Liang Zhang, Zhihui Li, Yan Yin, et al. Children glomerular huge rare disease [J]. Journal of clinical pathology and follow-up analysis. Pharmaceutical journal of pediatrics, 2014, 20 (12) : 11-15.doi: 10.13407 / j.carol carroll nki JPP. 1672-108 - x. 2014.12.004.
17. Shaofan Wang, Huiping Chen, Xiaodan Yao, et al. Clinical and pathological analysis of glomerular giant and rare disease [J]. Journal of nephrology and dialysis kidney transplantation,2009,18(04):329-333+357.

18. Gatto A, Ferrara P, Leoni C, Onesimo R, Zollino M, Emma F, Zampino G. Oligonephronia and Wolf-Hirschhorn syndrome: A further observation. Am J Med Genet A. 2018 Feb;176(2):409-414. doi: 10.1002/ajmg.a.38554.

19. Konomoto T, Kurogi J, Sawada H, Hisano S, Nunoi H. Osteogenesis imperfecta complicated with renal hypoplasia leads to chronic kidney disease. Pediatr Int. 2017 Mar;59(3):369-370. doi: 10.1111/ped.13221.

20. Miltényi M, Balogh L, Schmidt K, Detre Z, Hernády T, Czeizel A. A new variant of the acrorenal syndrome associated with bilateral oligomeganephronic hypoplasia. Eur J Pediatr. 1984 Apr;142(1):40-3. doi: 10.1007/BF00442589.

21. al Salloum AA, al Rasheed SA, al Husain MA, al Mugeiren MM, al Rikabi AS, al Sohaibani MO. Acrorenal syndrome associated with visual defect. Pediatr Nephrol. 1996 Dec;10(6):759-60. doi: 10.1007/s004670050211.

22. Kusuyama Y, Tsukino R, Oomori H, Kuribayashi K, Katayama H, Koike M, Saito K. Familial occurrence of oligomeganephronia. Acta Pathol Jpn. 1985 Mar;35(2):449-57. doi: 10.1111/j.1440-1827.1985.tb00587.x.

23. Park SH, Chi JG. Oligomeganephronia associated with 4p deletion type chromosomal anomaly. Pediatr Pathol. 1993 Nov-Dec;13(6):731-40. doi: 10.3109/15513819309048260.

24. Moerman P, van Damme B, Proesmans W, Devlieger H, Goddeeris P, Lauweryns J. Oligomeganephronic renal hypoplasia in two siblings. J Pediatr. 1984 Jul;105(1):75-7. doi: 10.1016/s0022-3476(84)80366-2.
